# Audit of essential knowledge of diabetes in patients with diabetes in Zimbabwe

**DOI:** 10.11604/pamj.2023.45.103.31770

**Published:** 2023-06-23

**Authors:** Åsa Ernersson, Esther Mufunda, Katarina Hjelm

**Affiliations:** 1 Department of Health, Medicine and Care, Linköping University, Linköping, Sweden,; 2 Department of Health Sciences, Zimbabwe Open University, Harare, Zimbabwe,; 3 Department of Public Health and Caring Sciences, Uppsala University, Uppsala, Sweden

**Keywords:** Diabetes mellitus, education, diabetes knowledge, self-care

## Abstract

**Introduction:**

in Zimbabwe, the organized diabetes education in the governmental health care system is limited, but the Diabetes Association has some educational activities in which persons with diabetes can participate. In this study, the purpose was to measure essential knowledge of diabetes and its management in persons with diabetes living in Zimbabwe.

**Methods:**

a cross-sectional descriptive study design was used to audit essential knowledge of diabetes and its management in persons with type 2 diabetes through a survey of 64 persons attending meetings at the Zimbabwe Diabetes Association. Both descriptive and analytic statistical methods were used.

**Results:**

in general respondents have acceptable knowledge of diabetes, whereas their knowledge of glycaemic control is low (45%), likewise their knowledge of how to manage medical treatment when ill. Knowledge concerning lifestyle-related issues was generally low. Respondents had limited knowledge about changes in blood glucose during physical activity (18%) and their knowledge about appropriate food for people with diabetes was low (67%). Most respondents were aware of the importance of regular examinations to avoid long-term complications related to diabetes (>87%) but their knowledge of how to prevent foot complications and perform daily preventive foot care was limited (73 %).

**Conclusion:**

there is limited knowledge of diabetes in Zimbabwean persons with type 2 diabetes even if they have participated in educational activities at the patient associations. This further supports the need for development of education for patients in health care which requires increased competence in the field among health care staff.

## Introduction

Diabetes mellitus is a major health problem with currently about 460 million adults aged 20-79 diagnosed or undiagnosed with the disease worldwide. In Africa, it is estimated that about 19 million people in the same age group had diabetes in 2019, but type 2 diabetes is predicted to increase soon to an even greater extent due to urbanization and the increasing age of the population [1]. In Zimbabwe the prevalence has increased rapidly during the last 40 years [2]. Health care for diabetes and diabetes-related complications is expensive and both the direct and the indirect costs are huge in Africa [3]. Self-care is a prerequisite for preventing and reducing the risk of developing acute and late complications related to diabetes mellitus. Patients´ knowledge and awareness of their diabetes, on the other hand, is crucial for self-care management [4]. A previous review indicates that self-management in sub-Saharan Africa is poor and therefore a serious threat to the health of individuals and the capacity of the health system [5]. There is an urgent need for improved awareness and management of diabetes to reduce the epidemiologic spread and devastating impact on health by diabetes and its complications [6]. Self-care management is affected by knowledge of the disease which in turn is influenced by the person´s beliefs about health and illness [7-10]. Lack of diabetes knowledge has been demonstrated in studies from Zimbabwe [11,12]. Zimbabwean adults with diabetes mellitus recruited from an outpatient diabetes clinic at a main referral hospital have been found to have low knowledge about diabetes, in terms of total knowledge, general knowledge and knowledge of insulin use. The main knowledge gaps were related to diet, insulin use and glycaemic control. In that study educational level was shown to be an independent determinant of total knowledge of diabetes and for insulin use knowledge [11]. Similar results were found by Mufunda *et al*. [12] in adult patients with diabetes attending meetings at the Zimbabwean Diabetes Association. In that study, female gender was associated with low general knowledge of diabetes. Health care professionals’ awareness of the patients´ knowledge gaps about diabetes makes it important to further study patients´ essential knowledge of diabetes and its management, to increase the conditions for diabetes care in these countries to develop and, in the long term, to reduce the patients´ unnecessary risk of complications related to the disease.

In Zimbabwe there is, in general, no organized diabetes care in the health care system. Diabetes care service is included in the national health care system and patients with more complex diabetes-related complications are expected to be referred up to the referral chain [13]. There is no national health insurance system in Zimbabwe. Public health care is free for pensioners and children under 5 years of age, meaning that most patients with type 2 diabetes who are not beneficiaries have to pay for diabetes care and/or drugs either in cash or through private health insurance. Drugs for diabetes treatment are usually very expensive. The patients visiting the main diabetes clinic at the highest level of the health care system, the central hospital, meet a general nurse who sometimes, while awaiting the answer to tests or preparing a visit to the physician, may give some general health education [12]. In the country there is no specialist training in diabetes care for nurses and only a few specialists in internal medicine have had education in diabetology. The lack of specialist knowledge of diabetes and diabetes care means that a patient remitted from the lower level to the highest level of care can meet a specialist in internal medicine with limited knowledge of diabetes, which might increase the risk of poor glycaemic control and diabetes-related complications despite medical diabetes treatment [13]. Another crucial factor Is the lack of proper monitoring of diabetes. It is essential to have strategies to achieve a healthy lifestyle and diet, screening services and improved accessibility to equipment and medical drugs [14]. In this study the purpose was to measure essential knowledge of diabetes and its management in persons with diabetes living in Zimbabwe.

## Methods

**Study design:** a cross-sectional descriptive study design was used in a survey to measure the respondent´s essential knowledge of diabetes and its management [15].

**Data collection:** through convenience sampling 64 participants (35 women and 29 men) attending meetings at the Zimbabwe Diabetes Association located in the capital, a non-governmental patient association, were recruited for this study. The Association members are persons with diabetes mellitus from different parts of Zimbabwe. The association supports individuals with diabetes by giving both material and information, usually referred from the hospital-based diabetes clinics as part of continued self-management. No diabetes specialists are employed by the Association, but occasionally specialist physicians, dieticians, psychologists, or diabetes nurses can be invited to come and give health education talks or to do free assessments of members and their families as part of ongoing support. Another reason to attend these meetings is the availability of insulin at lower prices than in public health care pharmacies, along with the chance to share experiences of the disease and to obtain information about diabetes and its management based on patients´ desires for information about topics such as diet, physical exercise, medication, self-care etc. Regular meetings are held monthly for about 2 hours per session [12]. The inclusion criteria were diagnosis with diabetes mellitus for at least one year, mentally sound to give informed consent and be conversant with either English or Shona (two of the three official languages in Zimbabwe) [12].

**Questionnaire:** all participants were asked to fill in a standardized self-report questionnaire including questions about essential knowledge of diabetes and its management, socio-demographic and diabetes-related background data. A registered nurse distributed the questionnaires when the respondents met at the Zimbabwe Diabetes Association. The total time required to complete the questionnaire was about an hour and, if necessary, questions were clarified and explained by the nurse [12].

**Essential knowledge of diabetes and its management:** to measure essential knowledge of diabetes and its management the Audit of Diabetes Knowledge (ADKnowl) was used [16]. ADKnowl contains of 23 item-sets (104 items) relating to treatment, sick days, hypoglycaemia, effects, foot care, and diet and food. Each item consists of a statement and respondents are asked to fill in whether the statement is true or false. There are two items-sets (7 items) that are to be answered only by patients using insulin and two item-sets (9 items) to be answered only by those who are treated with oral agents [16].

**Ethical considerations:** the Diabetes Association of Zimbabwe and the Medical Research Council of Zimbabwe gave permission to conduct this study (approval number MRC/B/287). Written informed consent was obtained from all the respondents and the study was performed in accordance with the Helsinki declaration [17].

**Data analysis:** descriptive statistics as mean (SD) were used to describe socio-demographic and diabetes-related background data. Categorical variables are presented with percentages and frequencies [18]. For the non-normally distributed variables, the non-parametric test Mann-Whitney U-test was used to compare knowledge of diabetes and its management between treatment regimens (oral agents and insulin/combination with insulin). The third group, diet, was excluded from the comparison due to the small sample (n =5). The Kruskal-Wallis test was used to compare the essential knowledge of diabetes between more than two groups, educational level and duration of diabetes. In this analysis no distinctions were made according to treatment regime. The significance level was set at p< 0.05. Data were analysed using SPSS, version 24 (SPSS Inc, IL, USA).

## Results

### Respondents´ socio-demographic and diabetes-related data

A total of 64 respondents filled in the questionnaires, and of these, 35 were women and 29 were men. The mean age of the respondents was 38(±17) years, and no significant difference was found in age between men and women (men 37± 15years, women 39± 19 years). Most of the respondents were married (44%) and education level varied with most of the respondents reporting secondary or tertiary/college-level as their education; while about two-thirds of the respondents were unemployed. The mean duration of diabetes was 11(±8) years and most of the respondents (62%) were on insulin or combination with insulin use regime. Twenty-seven percent of the respondents reported complications from diabetes, including eye (n = 7), heart (n = 1), lower extremity (n = 1) or other (n=8). The socio-demographic and diabetes-related characteristics of the respondents are summarized in Table 1. Of all 64 respondents, 44 had previous attended diabetes meeting in a diabetes class, 61% of the women (n = 27) and 39% of the men (n=17). About three quarters of the respondents on insulin regime had previous attended a diabetes class (n = 32) but just one third of the respondents on oral agents or diet regime (n = 9 vs 3) (data not shown) (Table 1).

**Table 1:** socio-demographic and diabetes-related characteristics of the respondents

Variable	Frequency n (%)
**Gender**	
Male	29 (45)
Female	35 (55)
**Marital status**	
Unmarried	24(38)
Married/cohabitant	28(44)
Divorced/separated	5(8)
Widow/er	7(11)
**Educational level**	
Primary school	6(9)
Secondary school	29(45)
Tertiary/college	17(27)
University <2 years	1(2)
University >2 years	11(17)
**Employment**	
Unemployed	40(63)
Gainfully employed	18(28)
Sick leave	2(3)
Retired	4(6)
**Treatment regime**	
Diet	5(8)
Oral agents	19(30)
Insulin or combination with insulin	40(62)
**Diabetes-related complications**	
Eye	7(11)
Lower extremity	1(2)
Heart	1(2)
Other	8(12)
**Duration of diabetes in years**	
≤3	7(11)
4-9	29(45)
≥10	28(44)

### General knowledge related to diabetes and glyceamic control

In general respondents have acceptable knowledge of diabetes with the exception of whether it is a good thing to have glucose in urine, where 45% answered incorrectly (Table 2). Respondents treated with oral agents do not have proper knowledge about the effects of their tablets and the importance of taking the tablets regularly (37% answered incorrectly). Respondents treated with insulin or in combination with insulin expressed limited knowledge about treatment with insulin when being ill or not eating well (90% answered the statement incorrectly) (data not shown). There was no significant difference in general knowledge between respondents who had attended diabetes classes and those who had not. In general, the knowledge about the meaning of what HbA1c reflects was low in all respondents (≥ 40% answered incorrectly), as well as in the treatment groups (Table 2). There was no difference in knowledge depending on whether respondents had participated in diabetes class or not. In general respondent have limited knowledge of symptoms of hypoglycaemia i.e feeling very thirsty or passing more urine than usual was incorrectly identified as common symptoms of hypoglycemia (61% respectively 41%). There was no significant difference between treatment groups considering knowledge of symptoms of hypoglycaemia. There was however a tendency for women to have significantly lower knowledge than men about glycaemic control (data not shown), as indicated in a few items of the ADK test. A larger number of women thought that too much glucose in the blood is a sign of hypoglycaemia (p = 0.011), said that confused thinking was not a common symptom of “hypos” (p = 0.02), and thought that it is advisable to take some insulin or tablets immediately when having symptoms of hypoglycaemia (p = 0.049) (Table 2).

**Table 2:** general knowledge related to diabetes and glycaemic control in all respondents. Knowledge concerning glycaemic control were generally limited in all respondents

	All respondents n= 64	Insulin or combination with insulin n=40	Oral agents n=19	Diets n=5
**Please consider each of the following statements about diabetes**	**All responses are given as % of incorrect responses**.			
Diabetes can be controlled with treatment	3	5	0	0
A little glucose in the urine is a good thing	45	48	42	40
Diabetes is likely to go away after a while	2	3	0	0
Stressful experiences can affect blood glucose levels	6	3	5	40
Exercises can help you improve or maintain blood glucose control	5	8	0	0
Achieving your ideal weight helps control diabetes	3	5	0	0
Blood glucose levels do not affect your chances of developing complications	14	18	5	20
**Please consider each of the following statements about hypoglycaemia:**				
in hypos there is little glucose in the blood	5	5	5	0
in hypos there is too much glucose in the blood	11	15	5	0
hypos can be caused by an increase in physical activity	17	20	11	20
**Please consider which of the following symptoms are commonly associated with “hypos”:**				
slurred speech	11	15	5	0
feeling very thirsty	61	53	68	0
sweating	11	15	5	0
dizziness	8	13	0	0
confused thinking	9	13	5	0
passing more urine than usual	41	43	26	80
**If you get the symptoms of a hypo, it is advisable to…**				
take some insulin or tablets immediately	17	18	21	0
eat or drink something which has sugar in it	8	8	11	0
rest for 10–15 minutes	19	23	16	0
test blood glucose level straight away	9	10	11	0
eat less food for the next meal	17	18	16	20
**The HBA1 (c) level in blood…**				
can show if you are getting hypos	9	85	100	100
reflects the average blood glucose over the past 6 to 8 weeks	40	45	32	40
reflects the average blood glucose over the past 6 to 8 days	45	40	58	40
reflects the average blood glucose level over the last 24 hours	56	58	58	40

No significant differences were found between respondents with insulin treatment or combination with insulin and respondents with oral agents. Respondents on diet regime were excluded from the comparison.

### Knowledge concerning lifestyle-related issues

In general, about one fifth of the respondents have no knowledge about the usual effect of physical activity on blood sugar levels. Persons with insulin treatment have limited knowledge about the effect of physical activity and the need of insulin and the relation to food intake, and about half of the respondents answered incorrectly (43-50%) (Table 3). Both men and women had limited knowledge about changes in blood glucose during physical activity, but men had even lower knowledge than women (p= 0.015). With a few exceptions, men thought that physical activity leaves blood glucose levels unchanged (97%) (data not shown). In general, despite treatment the respondent´s knowledge about appropriate food for persons with diabetes is low (Table 3). Regarding fruit and sugar content and the effect on blood glucose level, knowledge was also low (63% answered incorrectly), particularly in the insulin treatment group (73% answered the statement incorrectly). There is a significant difference between the treatment groups regarding the calorie content between margarines and spreads compared to butter, with the insulin treatment group knowing less. The same pattern is found concerning fat content in pastry and cakes as well as cheese and biscuits. Concerning diabetic products, most of the respondents thought that these can be eaten freely without leading to weight gain; those with insulin treatment answered incorrectly significant more than respondents on oral agents (58% vs 26%, p < 0.05).

**Table 3:** knowledge concerning lifestyle-related issues were generally limited in all respondents

	All respondents n= 64	Insulin or combination with insulin n=40	Oral agents n=19	Diets n=5
**The usual effect of physical activity is to…**	**All responses are given as % of incorrect responses**			
lower blood glucose level	11	5	11	60
raise blood glucose levels	11	8	11	40
increase glucose levels in urine	19	20	21	
leave blood glucose levels unchanged	16	18	11	20
improve the control of diabetes	6	8	0	20
**If you increase your physical activity it is advisable to1**				
take less insulin with the same food		43		
take the same amount of insulin with more food		50		
take more insulin with less food		13		
**Please consider each of the following statements about diet:**				
people with diabetes can eat food containing sugar as part of a high-fibre diet	67	68	63	80
sugary foods will have no effects on blood glucose levels	6	8	5	0
high-fibre food helps keep blood glucose levels steady	11	10	11	20
high-fat diet increases the risk of complications	6	10	0	0
special diabetic products can be eaten freely without leading to weight gain	47	58*	26	40
**Please consider each of the following statements about food:**				
the same sized portion of fish and white meat such as chicken contains less fat than red meat such as beef or pork	19	20	16	20
it is possible to eat too much protein	47	45	53	40
fried foods are usually high in fat	3	5	0	0
pastry and cakes are high in fat	52	63	32	40
cheese and biscuits are usually less fattening than puddings	41	40	37	60
all margarines and spreads have fewer calories than butter	50	63*	32	20
restricting use of salt can help to reduce high blood pressure	8	13	0	0
fresh fruit can be eaten freely with little effect on blood glucose	63	73	47	40
fresh, unsweetened fruit juice can be drunk freely with little effect on blood glucose levels	61	65	56	40
**If someone with diabetes smokes…**				
the risk of serious foot problems leading to amputation is increased	20	23	16	20
the risk of heart disease is increased	5	5	5	0
the risk of stroke is increased	6	5	5	20
it is no worse than for a person without diabetes	13	13	16	0
it can be a good way of keeping their weight down	9	8	16	0
**Alcoholic drinks generally…**				
lower blood glucose levels after a few hours	69	60	84	80
raise blood glucose levels	22	20	26	20
have no calories	20	25	11	20
1Item answered by respondents with insulin treatment or oral agents in combination with insulin treatment * p≤0.05				

Significant differences were found between respondents with insulin treatment or combination with insulin and respondents with oral agents. Respondents on diet regime were excluded from the comparison.

In general, most of the respondents believe that people with diabetes can eat food with sugar as part of high-fibre diets (almost 70%). Knowledge about fresh fruit and blood glucose differed between those who had attended diabetes classes and those who had not. Seventy-three per cent of those who had attended diabetes class thought that fresh fruit can be eaten freely without having any effect on blood glucose level, while 42% of those who had not attended diabetes class thought the same (p 0.022). There was also a significant difference (p=0.047) concerning margarine and spread compared to butter, with those who had attended diabetes class answering incorrectly to a higher extent. Knowledge of the long-term effect on blood glucose level of drinking alcohol was limited, although more so in those treated with oral agents (about 80%). The same was seen in the group treated with diet. About one-fifth of the respondents were not aware of the relation between smoking and the risk of developing serious foot complication leading to amputation (Table 3).

### Knowledge about identifying complications

The respondents were in general aware of the importance of regular examinations to avoid long-term complications related to diabetes. However, their knowledge of how to prevent foot complications and perform daily preventive footcare was limited. No significant difference was found between respondents who had attended diabetes class and those who did not, nor were there any gender differences (data not shown).

## Discussion

This study found a general lack of knowledge of diabetes, with the greatest knowledge gap concerning diet related to diabetes and the relation to blood glucose. The diet recommended is suitable for all people irrespective of the disease and thus not specially adjusted to persons diagnosed with diabetes [4]. Even those who stated they had participated in diabetes classes still showed knowledge gaps. Respondents with insulin treatment stated to a higher extent than others that they attended diabetes classes, but still had limited knowledge about diet. This verifies and supports the previous results from the study by Mufunda *et al*. [11], where major knowledge gaps were found related to diet and glycaemic control, and as indicated in qualitative studies on beliefs about health and illness [8,10].

In this study respondents seems to know about symptoms of diabetes, but they are not able to differentiate between symptoms related to hyper and hypoglycaemia. The key symptoms of hyperglycaemia (feeling thirsty and passing more urine than usual and having glucose in the urine) were mistaken for symptoms of hypoglycaemia by about half of the respondents. This is a sign of lack of knowledge of when to react to symptoms related to increased risk of developing complications of diabetes. Thus, the results of this study show that the respondents in general had limited knowledge of diabetes and its complications. A study by Machingura *et al*. [19] measuring the prevalence of nephropathy and related factors in patients with diabetes visiting an outpatient clinic in Harare, Zimbabwe, found that more than one third of the 344 patients included had increased levels of albuminuria and hence nephropathy. The high incidence of nephropathy in patients with diabetes is serious as it will have major consequences for both the patient´s costs and healthcare costs in the future. Self-care management to prevent foot ulcer is one of the cornerstones in diabetes management [4]. As in a study from Uganda [20], focusing on beliefs about health and illness in relation to foot care, similar results were found, with lack of knowledge about daily management of the foot and prevention of diabetic foot ulcers. This implies an increased risk of developing severe diabetes-related complications and the patients urgently need to be supported with diabetes education to increase their awareness of the importance of daily foot care to prevent diabetes-related foot complications.

Even if they state that they have attended diabetes class, the results are still contradictory, e.g. the relation between diabetes and diet or physical activity is not recognized, further supporting the need for diabetes education. A previous study has shown a relation between diabetes education and knowledge [21], although the results are not comparable since other instruments measuring knowledge were used. Further, it is not known in our study what kind of education the respondents refer to when talking about attending diabetes classes. Most likely they have attended sessions run by the diabetes association, since there is no organized diabetes education in health care [13]. Nor do we know anything about the content or the quality of the education given by the diabetes association. On the other hand, patients attending the diabetes association´s activities have probably sought support mainly to be able to get access to medications, as there is often lack of available medications (mainly insulin) at the hospital, but there are also other reasons, for example, a quest for knowledge, which might attract more diabetes patients in general. There are probably a huge number of patients who do not attend these activities at all and thus have even less chance of obtaining medications and knowledge. This might increase the burden of the diabetes pandemic even more, both on the individual and in socioeconomic terms [2]. Thus, there is an urgent need for development in diabetes education, both in general health care and in education delivered by non-governmental organizations such as the diabetes association. Further, this also requires education of health care staff to be able to support the patients in their self-care. In Zimbabwe there is a lack of specialist training for nurses in diabetes care, and the number of physicians specialized in diabetes management is limited [13]. Further, the content of the training programmes for both nurses and physicians also needs to be improved. To develop cost-effective and optimal diabetes care in a country with limited resources, not only educational efforts are needed but also systemic and structural conditions need to be considered in order to promote health and to prevent costly consequences of DM [8].

**Limitations:** the strength of the study is the use of a validated and reliable instrument, ADKnowl [16]. Another strength is that the results are supported by a previous study [11], although the persons investigated in this study might be a positive selection since their educational level is higher than in the previous study [11]. Another possible selection bias may be that the participants also had a greater interest in learning about diabetes since they had voluntarily come to the association meetings offering activities such as diabetes education and enables an environment to share disease experiences with others. On the other hand, another reason for attending this association´s meetings is to get access to proper medical treatment that often is lacking in the governmental hospital where they usually have their main diabetes management. However, the results of this study indicate that even if the patients participate in these meetings and get education, they still have limited knowledge. A study sample from the general population with diabetes might had given different results. The sample size (N=64) can be seen as limited but due to the inclusion criteria all that participated in the meetings of the studied association during three occasions during the study period were asked to participate and agreed.

The knowledge might be influenced by the constructions of the questions in the survey, since the main focus is on symptoms related to hypoglycaemia instead of hyperglycaemia. Later in the instrument the relation between blood glucose and long-term complications is studied, which might have misled the participants. It is also evident that the respondents do not understand the relation between diet, physical activity and blood glucose. With limited knowledge in general, there is a great need of proper patient education because the education given at the association is not sufficient and there is a serious lack of patient education at the hospital. In the instrument there are also questions about foot care and use of appropriate shoes, and the question is what knowledge of foot care and access to proper shoes the general population in Zimbabwe has. In a previous study from Tanzania the prevalence of foot problems was higher than among Swedes with diabetes [22]. This was related to limited knowledge of diabetes foot care among Tanzanians and a cultural tradition of walking barefoot and not protecting the feet [22]. Since Tanzania and Zimbabwe are close to each other both geographically and culturally, the same circumstances can probably be found in Zimbabwe. A previous study from Uganda has also shown a delay in the health care process due to patients´ beliefs that may have deleterious consequences for life and limb in persons with diabetic foot ulcers [20].

## Conclusion

In conclusion, there is limited knowledge of diabetes in Zimbabwean persons with type 2 diabetes even if they have participated in educational activities at the patient associations. This further supports the need for the development of patient education in health care, which requires increased competence in this field among health care staff.

### What is known about this topic

- *Patients´ knowledge and awareness of their diabetes is crucial for self-care management and hence, for preventing and reducing the risk of developing acute and late complications related to diabetes mellitus;*
- *Persons with diabetes in Zimbabwe have knowledge gap related to diet, insulin use and glycaemic control*.

### What this study adds

- *There is limited knowledge of diabetes in Zimbabwean persons with type 2 diabetes even if they have participated in educational activities at the patient associations;*
- *This study further supports the need for the development of patient education in the governmental health care, which requires increased competence in this field among health care staff*.
